# Micro-photoluminescence of GaAs/AlGaAs triple concentric quantum rings

**DOI:** 10.1186/1556-276X-6-569

**Published:** 2011-10-31

**Authors:** Marco Abbarchi, Lucia Cavigli, Claudio Somaschini, Sergio Bietti, Massimo Gurioli, Anna Vinattieri, Stefano Sanguinetti

**Affiliations:** 1 L.E.N.S. and Dipartimento di Fisica, Universitá di Firenze, Via Sansone 1, I-50019, Sesto Fiorentino, Italy; 2 L-NESS and Dipartimento di Scienza dei Materiali, Universitá di Milano Bicocca, Via Cozzi 53, I-20125, Milano, Italy

## Abstract

A systematic optical study, including micro, ensemble and time resolved photoluminescence of GaAs/AlGaAs triple concentric quantum rings, self-assembled via droplet epitaxy, is presented. Clear emission from localized states belonging to the ring structures is reported. The triple rings show a fast decay dynamics, around 40 ps, which is expected to be useful for ultrafast optical switching applications.

## 

Self-assembled semiconductor quantum nanostructures are currently deeply investigated because of their potentiality as building block for novel quantum optical and optoelectronic devices [1-4]. Several promising applications in these fields, such as single photon source for quantum cryptography [1], quantum bits [4], or quantum logical elements [3,4], require the coherent manipulation of the carrier population in the adjacent quantum nanostructures. Within this scenario, quantum rings have several interesting peculiarities. First, unlike quantum dots, the quantum ring ground state total angular momentum changes from zero to non zero by increasing the magnetic field [5,6]. This also results in a peculiar energy dispersion of the excitons for different ring radius. Second, since charge tunneling between states of different angular momentum is strongly suppressed by selection rules, double concentric quantum rings eventually offer the control of effective coupling of direct-indirect excitons [7]. The possibilities to control the coupling between different rings is indeed of the utmost relevance in the research of semiconductor-based quantum computational devices.

The recent success in self-assembly of single and multiple concentric quantum rings by Droplet Epitaxy (DE) [8,9] has provided suitable semiconductor nanostructures for the investigation of these fundamental physical effects.

Droplet epitaxy [10,11] is a flexible growth method, based on Molecular Beam Epitaxy (MBE), which allows for the fabrication of a large variety of three dimensional nanostructures with different geometries, such as quantum dots [12], quantum dot molecules [13], quantum rings [8,14] and coupled disk-ring structures [15]. The intrinsic design flexibility of the DE is permitted mainly by the splitting in time of the III-column and V-column element supply. This allows an independent choice, for each of the two elements, of specific growth conditions.

We have recently reported the possibility to fabricate multiple (up to five) concentric quantum ring structures [9]. Here we present the detailed micro and time resolved photoluminescence characterization of the optical and electronic properties of one of such DE quantum nanostructures, made by three concentric quantum ring (TCQR).

The TCQR fabrication was performed following the recipe reported in Ref. [9] in a conventional GEN II MBE system on GaAs (001) substrates. After the growth of a 750 nm-thick GaAs buffer layer and of a 200 nm-thick Al_0.3_Ga_0.7_As barrier layer at 580°C, the substrate temperature was decreased to 350°C and the As valve closed. At the same temperature a Ga molecular beam equivalent to the formation of 10 ML of GaAs in presence of As was supplied to the substrate surface, leading to the formation of numerous nearly hemispherical Ga droplets. Their average diameter and height were around 80 nm and 35 nm, respectively, while the density was estimated to be around 8 × 10^8 ^cm^-2^. After the formation of Ga droplets, the substrate temperature was decreased to 250°C and an As BEP of 8 × 10^-7 ^Torr was supplied for 20 seconds for the partial crystallization of the original droplets into GaAs. Finally the substrate temperature was increased to 300°C and the sample surface was irradiated by the same As BEP (8 × 10^-7 ^Torr) for 20 minutes, in order to ensure the complete reaction of metallic Ga with As. At the end of the procedure, clear concentric triple rings structures with good rotational symmetry appeared, with inner, middle and outer ring diameters of around 80 nm, 140 nm and 210 nm respectively, while heights were around 7 nm for the inner rings, 4 nm for middle rings and 3 nm for the outer rings. Atomic force microscopy (AFM) image and the corresponding cross sectional height profile are reported in Figure 1). The TCQRs showed an elongation of around 11% along the $\left[0\bar{1}1\right]$ direction which might be due to the anisotropic diffusion of Ga on GaAs (001) surface [16,17]. The inner ring in TCQRs nearly showed the same diameter respect to the original Ga droplet and the density of the TCQRs structures was equal to that of the original droplets, confirming that all Ga droplets were transformed into GaAs triple rings at the end of the procedure. After the last As pulse at 300°C, the sample was annealed under As flux at T = 350°C for 10 min. Subsequently the structures were capped with Al_0.3_Ga_0.7_As for carrier confinement (20 nm at 350°C by migration enhanced epitaxy [18], 40 nm by standard MBE at 580°C). A thin layer of GaAs grown at 580°C capped the structure. The sample was then annealed at 650°C for 1 hour in the MBE chamber, to improve the quality of the structure, just after the growth [19,20].

**Figure 1:**
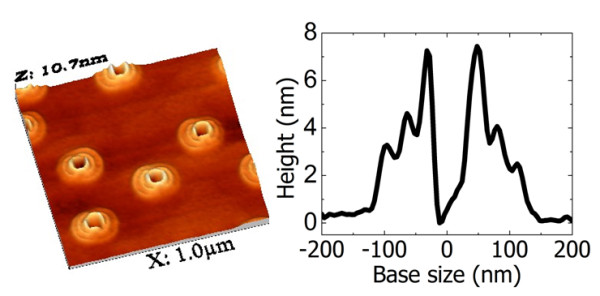
**Left Panel: AFM image of the as grown sample before AlGaAs capping showing TCQRs; Right Panel: AFM profile of a single TCQR**.

The ensemble optical emission of TCQR structures is shown in Figure 2. The photoluminescence was measured at T = 15 K and excited with a green laser (*λ*_exc _= 532 nm) at an excitation power density P_exc _= 10 W/cm^2^. A clear emission peak is visible at *E*_A _= 1.56 eV (band A), above the excitonic GaAs signature at 1.519 eV, with a full width at half maximum (FWHM) of 30 meV. A second emission (band B) appears just below the AlGaAs barrier. As shown in Ref. [21] band B is related to a thin GaAs quantum well-like layer which develops during the growth interruption times because of Ga migration from the droplet in a low As background.

**Figure 2:**
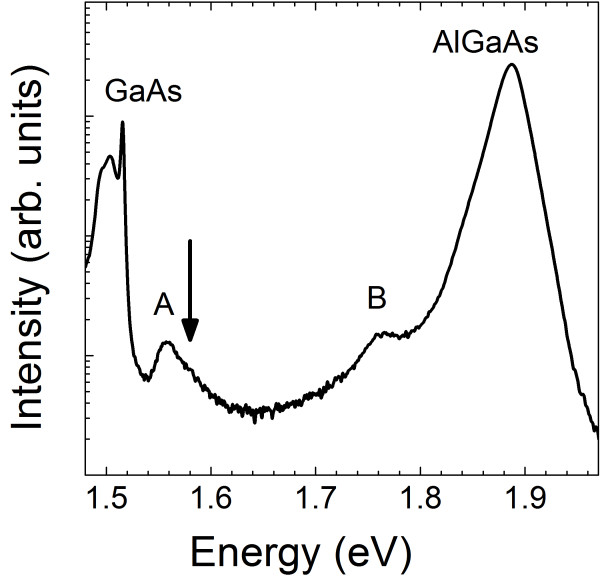
**Low temperature ensemble PL of the TCQR sample**.

The micro-PL spectra, recorded with a spot diameter of ≈ 1 *μ*m, show a spatial modulation of the spectra, in the 1.530-1.580 eV window, thus supporting the presence of emission from localized structures (Figure 3). The TCQR micro-PL spectra show a broad emission, with FWHM of 15 meV. In our spectra, we expect to simultaneously observe the emission from around ten TCQRs, given the relatively high TCQR density (*ρ *= 8 × 10^8 ^cm^-2^), and the large spatial extension of the outer ring structures (≈ 200 nm). The spectral differences from site to site should come from a local variation of the size distribution of TCQRs. Even if a limited number of structures is collected from our apparatus, no sharp emission lines from single TCQRs is detected, although this would be expected in the case of DE quantum nanostructures [22].

**Figure 3:**
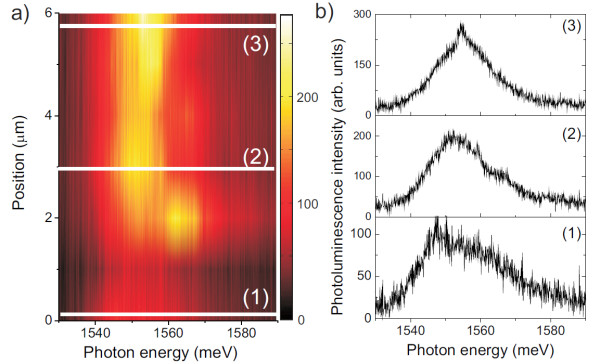
**Left panel: Dependence on sample position of the micro-PL emission of the TCQRs**. The spectra were measured at T = 15 K, *λ*_exc _= 532 nm and *P*_exc _= 5 W/cm^2^. The color scale is in arbitrary units. Right panel: Micro-PL spectra at positions corresponding to white lines on the scan.

Given the large extension of the ring wave-function, the line broadening is indeed expected to be enhanced in rings respect to dots, due to: i) Confinement energy fluctuations due to size disorder along the ring [23]; ii) The wire-like density of states of the rings in conjunction with state filling effects. All these effects may contribute to the broad emission from the rings. As a matter of fact, broad emission PL bands (of the order of few meV) from DE-ring structures are reported in literature [23,24]. However line broadening in our TCQRs is of the order of 10 meV and a major contribution stemming from spectral diffusion via quantum confined Stark effect induced by charge defects in the TCQR environment [25] is very likely.

Further insights into the emission properties of TCQR may be found by performing state filling experiments. The dependence of the PL of a single TCQR on P_exc _is shown in Figure 4. As the power density increases, the PL intensity grows superlinearly. This effect has been attributed in DE nanostructures to the saturation of non-radiative recombination channels in the barrier active during photogenerated carrier diffusion and capture [19]. At the same time, the TCQR PL spectrum shows a high energy shoulder (see band A2, Figure 4), whose relative intensity increases with P_exc_, located 20 meV above the fundamental peak.

**Figure 4:**
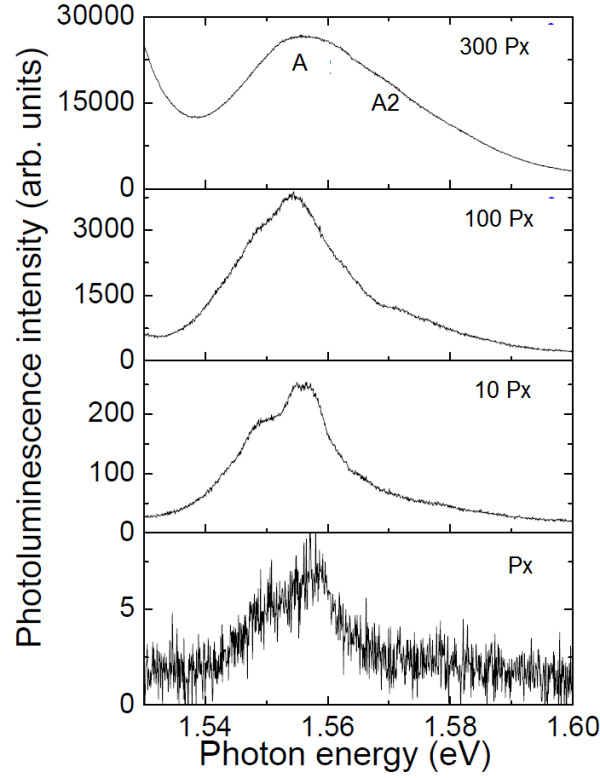
**Power dependence of micro-PL spectra of TCQRs**. The excitation laser power P = _exc _labels each spectrum (Px = 5W/cm^2^.

In order to attribute these lines, we performed electronic structure calculations following the method outlined in Refs. [19] within the effective mass approximation. We used the same materials parameters reported in Ref. [14] for GaAs and Al_0.3_Ga_0.7_As. In the calculations, the potential for quantum confinement was derived by the actual shape of a randomly chosen TCQR, measured by AFM and by imposing a cylindrical symmetry to the confining potential. In this framework we took into account the quantized motion of two-dimensional degrees of freedom, i.e., the radial motion, specified by the principal quantum number *n*, and the rotational motion, specified by the angular momentum *J*. The calculated main contributions to the optical transition spectra result from the recombination of an electron-hole pairs characterized by the same quantum numbers and indicated by 〈*nJ*〉. The main differences in energy pertain to states with different *n*. For each value of *n*, a band of closely spaced transitions (differing by *J*) is formed. The predicted TCQR ground state (〈00〉) transition energy is *E*_00 _= 1.580 eV. The ground state wavefunction is completely localized in the inner ring (Figure 5). The first radial excited state (〈10〉) is located 26 meV above the ground state (*E*_10 _= 1.606). The excited state wavefunction is, as for the 〈00〉 case, localized in the inner ring volume, but showing a *p*-like structure. At higher *n *values (see Figure 5), it is possible to find states fully localized in the second ring (*n *= 4) and in the third ring (*n *= 7).

**Figure 5:**
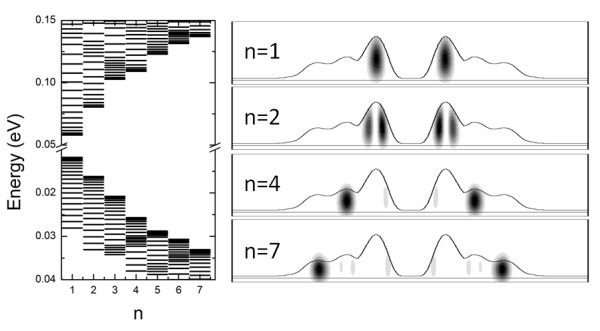
**Left panel: Single-carrier energy levels in TCQR**. Quantization energies for an electron and a heavy hole with the seven lowest radial quantum numbers n and various angular momenta (up to 20) are presented. Right panel: Cross-sectional imaging of electron probability density in TCQR for n = 1, 2, 4 and 7 with J = 0. The line represents the potential of confinement used for calculation.

The predicted 〈00〉 transition energy *E*_00 _lies well within the A line bandwidth. This allows us to safely attribute band A to the ensemble emission from the TCQR ground states. On the other side, the energy difference between A and A2 bands closely matches the energy difference *E*_10 _- *E*_00 _= 26 meV. In addition, the P_exc _behavior of band A and A2 is very similar to that shown by quantum dot ensembles where the additional band appearing at high P_exc _is attributed to state filling and radiative emission from the excited states. According to this similarity and our calculation, we attribute band A2 to the 〈10〉 transition. It should be stressed that the linked dynamics between 〈00〉 and 〈10〉 transition stems from the fact that the two states are localized within the same ring, thus showing an agreement with what has been found in single ring structures [14]. In fact, states which belong to different rings, as shown in Double Concentric Quantum Ring Structures [23], show a decoupled dynamics.

Time resolved ensemble PL of the TCQR sample is reported in Figure 6. TCQRs show an extremely fast decay time *τ_D _*= 40 ps, to be compared with the usual DE quantum dot and quantum ring values, which range between 300 and 500 ps [22,23,26]. The fast optical response cannot be likely attributed to non radiative processed arising from the defected TCQR barrier, because of the much longer decay times of the GaAs (*τ*_GaAs _= 300 ps) and of the Al_0.3_Ga_0.7_As barrier (*τ*_AlGaAs _= 200 ps), measured in the same sample at the same conditions (Figure 6), and denoting the good quality of the grown layers. The puzzling short *τ_D _*should be tentatively attributed to the outcome of an intrinsic decay mechanism in the TCQR such as strong electron-hole overlap and large transition dipole matrix element, even if the presence of parasitic channels inside the TCQR cannot be ruled out and it possibly plays a role.

**Figure 6:**
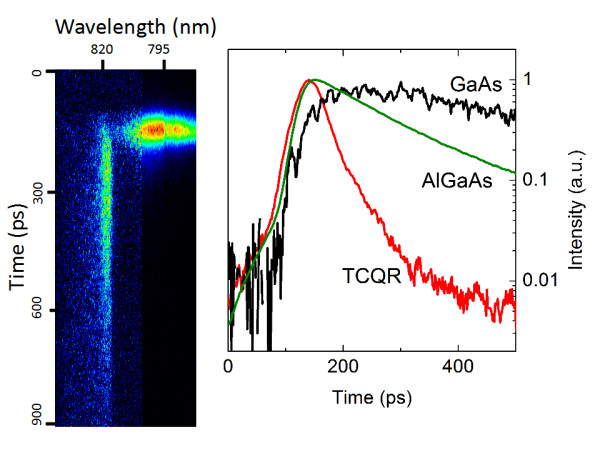
**Left panel: Temporally and spectrally resolved images of the TCQR sample**. GaAs and TCQR emissions are labelled. Right panel: Time resolved PL traces of the GaAs, AlGaAs and TCQR emissions.

In conclusion, we presented the optical characterization, also time resolved, of a single and of an ensemble TCQRs. The TCQRs are optically active, with a band centered around 1.56 eV. The theoretical calculations, performed in the effective mass approximation, are in good agreement with the experimental results. The three rings in the structure are able to quantum confine the electronic wavefunction within each individual ring. The TCQR show an ultrafast carrier dynamics, with a decay time of only 40 ps. TCQR are then promising self-assembled materials for ultrafast optical switches for high-bit-rate operations.

## Competing interests

The authors declare that they have no competing interests.

## Authors' contributions

MA and LC performed the photoluminescence measurements and analysed the data. CS and SB fabricated the sample. MG and AV designed the experiment and analysed the data. SS designed the experiment, analysed the data and coordinated the study. All authors drafted and approved the manuscript.

## References

[B1] Michler P Kiraz A Becher C Schoenfeld WV Petroff PM Zhang L Hu E Imamoglu A A Quantum Dot Single-Photon Turnstile Device Science 2000 290 2282 10.1126/science.290.5500.2282 11125136

[B2] Stevenson RM Thompson RM Shields AJ Farrer I Kardynal BE Ritchie DA Pepper M Quantum Dots as a Photon Source for Passive Quantum Key Encoding Phys Rev B 2002 66 081302(R)

[B3] Li X Wu Y Steel D Gammon D Stievater TH Katzer DS Park D Piermarocchi C Sham LJ An All-Optical Quantum Gate in a Semiconductor Quantum Dot Science 2003 301 809 10.1126/science.1083800 12907794

[B4] Solinas P Zanardi P Zanghi N Rossi F Semiconductor-Based Geometrical Quantum Gates Phys Rev B 2003 67 121307(R)

[B5] Lorke A Luyken RJ Govorov AO Kotthaus JP Petroff PM Spectroscopy of nanoscopic semiconductor rings Phys Rev Lett 2000 84 2223 10.1103/PhysRevLett.84.2223 11017249

[B6] Fuhrer A Lüscher S Ihn T an K Ensslin TH Wegscheider W Bichler M Energy spectra of quantum rings Nature 2001 413 822 10.1038/35101552 11677600

[B7] Dias da Silva LGGV MVillas-Bôas J Ulloa SE Tunneling and optical control in quantum ring molecules Phys Rev B 2007 76 155306

[B8] Mano T Kuroda T Sanguinetti S Ochiai T Tateno T Kim J Noda T Kawabe M Sakoda K Kido G Koguchi N Self-Assembly of Concentric Quantum Double Rings Nano Lett 2005 5 3 425 10.1021/nl048192+ 15755088

[B9] Somaschini C Bietti S Koguchi N Sanguinetti S Fabrication of multiple concentric nanoring structures Nano Lett 2009 9 10 3419 24 10.1021/nl901493f 19764709

[B10] Koguchi N Takahashi S Chikyow T New MBE growth method for {InSb} quantum boxes J Cryst Growth 1991 111 688 10.1016/0022-0248(91)91064-H

[B11] Koguchi N Ishige K Growth of GaAs epitaxial microcrystals on an S-terminated GaAs substrate by successive irradiation of Ga and As molecular beams Jpn J Appl Phys 1993 32 2052 10.1143/JJAP.32.2052

[B12] Watanabe K Koguchi N Gotoh Y Fabrication of GaAs Quantum Dots by Modified Droplet Epitaxy Jpn J Appl Phys 2000 39 2 L79 10.1143/JJAP.39.L79

[B13] Yamagiwa M Mano T Kuroda T Tateno T Sakoda K Kido G Koguchi N Minami F Self-Assembly of Laterally Aligned {GaAs} Quantum Dot Pairs Appl Phys Lett 2006 89 113115 10.1063/1.2354007

[B14] Kuroda T Mano T Ochiai T Sanguinetti S Sakoda K Kido G Koguchi N Optical transitions in quantum ring complexes Phys Rev B 2005 72 20 205301

[B15] Somaschini C Bietti S Sanguinetti S Koguchi N Fedorov a Self-assembled GaAs/AlGaAs coupled quantum ring-disk structures by droplet epitaxy Nanotechnology 2010 21 12 125601 10.1088/0957-4484/21/12/125601 20182013

[B16] Ohta K Kojima T Nakagawa T Anisotropic surface migration of Ga atoms on GaAs (001) J Crystal Growth 1989 95 71 10.1016/0022-0248(89)90354-0

[B17] Somaschini C Bietti S Fedorov A Koguchi N Sanguinetti S Concentric Multiple Rings by Droplet Epitaxy: Fabrication and Study of the Morphological Anisotropy Nanoscale Research Letters 2010 5 1865 1867 10.1007/s11671-010-9699-6 21170420 PMC2995438

[B18] Horikoshi Y Kawashima M Yamaguchi H Migration-Enhanced Epitaxy of GaAs and AlGaAs Jpn J Appl Phys 1988 27 2 169

[B19] Sanguinetti S Watanabe K Kuroda T Minami F Gotoh Y Koguchi N Effects of post-growth annealing on the optical properties of self-assembled GaAs/AlGaAs quantum dots J Cryst Growth 2002 242 3-4 321 10.1016/S0022-0248(02)01434-3

[B20] Mantovani V Sanguinetti S Guzzi M Grilli E Gurioli M Watanabe K Koguchi N Low density GaAs/AlGaAs quantum dots grown by modified droplet epitaxy J Appl Phys 2004 96 8 4416 10.1063/1.1791756

[B21] Somaschini C Bietti S Fedorov a Koguchi N Sanguinetti S Growth Interruption Effect on the Fabrication of GaAs Concentric Multiple Rings by Droplet Epitaxy Nanoscale Research Letters 2010 5 12 1897 1900 10.1007/s11671-010-9752-5 21170414 PMC2991240

[B22] Kuroda T Sanguinetti S Gurioli M Watanabe K Minami F Koguchi N Picosecond nonlinear relaxation of photoinjected carriers in a single GaAs/Al0.3Ga0.7As quantum dot Phys Rev B 2002 66 12 121302(R)

[B23] Sanguinetti S Abbarchi M Vinattieri A Zamfirescu M Gurioli M Mano T Kuroda T Koguchi N Carrier dynamics in individual concentric quantum rings: Photoluminescence measurements Phys Rev B 2008 77 12 125404

[B24] Abbarchi M Mastrandrea C Vinattieri A Sanguinetti S Mano T Kuroda T Koguchi N Sakoda K Gurioli M Photon antibunching in double quantum ring structures Phys Rev B 2009 79 8 085308

[B25] Abbarchi M Troiani F Mastrandrea C Goldoni G Kuroda T Mano T Sakoda K Koguchi N Sanguinetti S Vinattieri A Gurioli M Spectral diffusion and line broadening in single self-assembled GaAs/AlGaAs quantum dot photoluminescence Appl Phys Lett 2008 93 16 162101 10.1063/1.3003578

[B26] Abbarchi M Gurioli M Sanguinetti S Zamfirescu M Vinattieri A Koguchi N Recombination lifetime of single GaAs/AlGaAs quantum dots Physica Stat Sol (c) 2006 3 11 3860 3863 10.1002/pssc.200671578

